# Immunohistochemistry as a tool to identify *ELP1*-associated medulloblastoma

**DOI:** 10.1007/s00401-022-02409-4

**Published:** 2022-02-23

**Authors:** Arnault Tauziède-Espariat, Léa Guerrini-Rousseau, Alexandre Perrier, Jacob Torrejon, Flavia Bernardi, Pascale Varlet, Lauren Hasty, Olivier Delattre, Kévin Beccaria, Alice Métais, Olivier Ayrault, Fabrice Chrétien, Franck Bourdeaut, Christelle Dufour, Julien Masliah-Planchon

**Affiliations:** 1 Department of Neuropathology, GHU Paris-Psychiatry and Neuroscience, Sainte-Anne Hospital, 1, rue Cabanis, 75014 Paris, France; 2 Institut de Psychiatrie Et Neurosciences de Paris (IPNP), UMR S1266, INSERM, IMA-BRAIN, Paris, France; 3 Université de Paris, Paris, France; 4 Department of Children and Adolescents Oncology, Gustave Roussy, Paris Saclay University, Villejuif, France; 5 Team “Genomics and Oncogenesis of Pediatric Brain Tumors”, INSERM U981, Gustave Roussy, Paris Saclay University, Villejuif, France; 6 Laboratory of Somatic Genetics, Curie Institute Hospital, Paris, France; 7 Institut Curie, PSL Research University, CNRS UMR, INSERM, 91898 Orsay, France; 8 Université Paris Sud, Université Paris-Saclay, CNRS UMR 3347, INSERM U1021, 91898 Orsay, France; 9 Department of Pediatric Neurosurgery, Necker Hospital, APHP, Université Paris Descartes, Sorbonne Paris Cite, 75015 Paris, France; 10 SIREDO Center Care, Innovation, Research in Pediatric, Adolescent and Young Adult Oncology, Curie Institute and Paris Descartes University, Paris, France

The occurrence of a mutation in a cancer predisposition gene has been estimated to account for more than 40% of the medulloblastoma (MB), SHH-activated [3, 4]. Fourteen percent of them have been reported with bi-allelic alterations of *ELP1*, a tumor-suppressor gene being currently the most frequent to predispose to MB [4]. The *ELP1* gene encodes for the protein ELP1 which is a component of the elongator complex, a six-subunit protein complex (ELP1-6) implicated in neurogenesis [1, 2]. The bi-allelic inactivation of *ELP1* results from the combination of a germline alteration and a loss of chromosome 9q [4]. The aim of our study was to evaluate the sensitivity and specificity of ELP1 immunostaining to detect *ELP1*-associated MB.

Our study included a total of 132 DNA-methylation profiled MB: 57 SHH-activated (aged from 0- to 18-year-old), 15 WNT-activated, 30 group 3, and 30 group 4. We performed immunohistochemistry (IHC) for the ELP1 antibody (clone 6G9; 1:50 dilution; Sigma-Aldrich; Bromma, Sweden) on 3 µm-thick sections of formalin-fixed, paraffin-embedded tissue samples, performed on an Omnis automate. Tumoral molecular analysis of *ELP1* was conducted with a custom Next-Generation Sequencing (NGS) panel (Supplementary table S1). The library was prepared with the SureSelect XT-HS according to the manufacturer’s protocol (Agilent) and sequenced on an Illumina NovaSeq 6000. The sequence of all coding exons of *ELP1* (NM_003640.4) and *PTCH1* (NM_000264.3) and the loss of the heterozygosity (LOH) status of chromosome 9q were analyzed afterward. Proteomic has been quantified by a data-independent acquisition method following the same protocol as in [4]. We selected a proteome dataset composed of 16 MB, SHH-activated with five samples showing the *ELP1* pathogenic variation identified by genome and proteome techniques and previously reported in [4]. Finally, we tested by immunohistochemistry other pediatric tumor types of the posterior fossa (47 ependymomas, group A, 15 ependymomas, group B, 10 embryonal tumors with multilayered rosettes, 10 atypical teratoid and rhabdoid tumors, 10 central nervous system tumors with *BCOR* internal tandem duplication, and 10 pilocytic astrocytomas).

A complete loss of cytoplasmic ELP1 staining in all tumor cells (with intra-tumoral vessels as a positive internal control) was observed in 12/57 (21%) of MB, SHH-activated (Fig. 1a–c), and was preserved in all other MB subgroups (Fig. 1d) and in other tumor types (Supplementary Fig. 1). Molecular analyses revealed the presence of bi-allelic *ELP1* alterations (Table 1 for details) in 11/12 MB, SHH-activated, where ELP1 stained negatively. Thus, the sensitivity and the specificity of the IHC were evaluated as 99% (121/122) and 100% (11/11), respectively, in MB. Interestingly, for the unique discordant case, proteomic analyses revealed concordant downregulated levels of ELP1 (Supplementary Fig. 2). From a molecular perspective, this MB harbored a chromosome 9q copy-neutral LOH (confirmed by FISH analysis of chromosome 9) but the sequencing analysis failed to reveal any additional nucleotidic or copy number alteration at the *ELP1* locus.

**Fig. 1:**
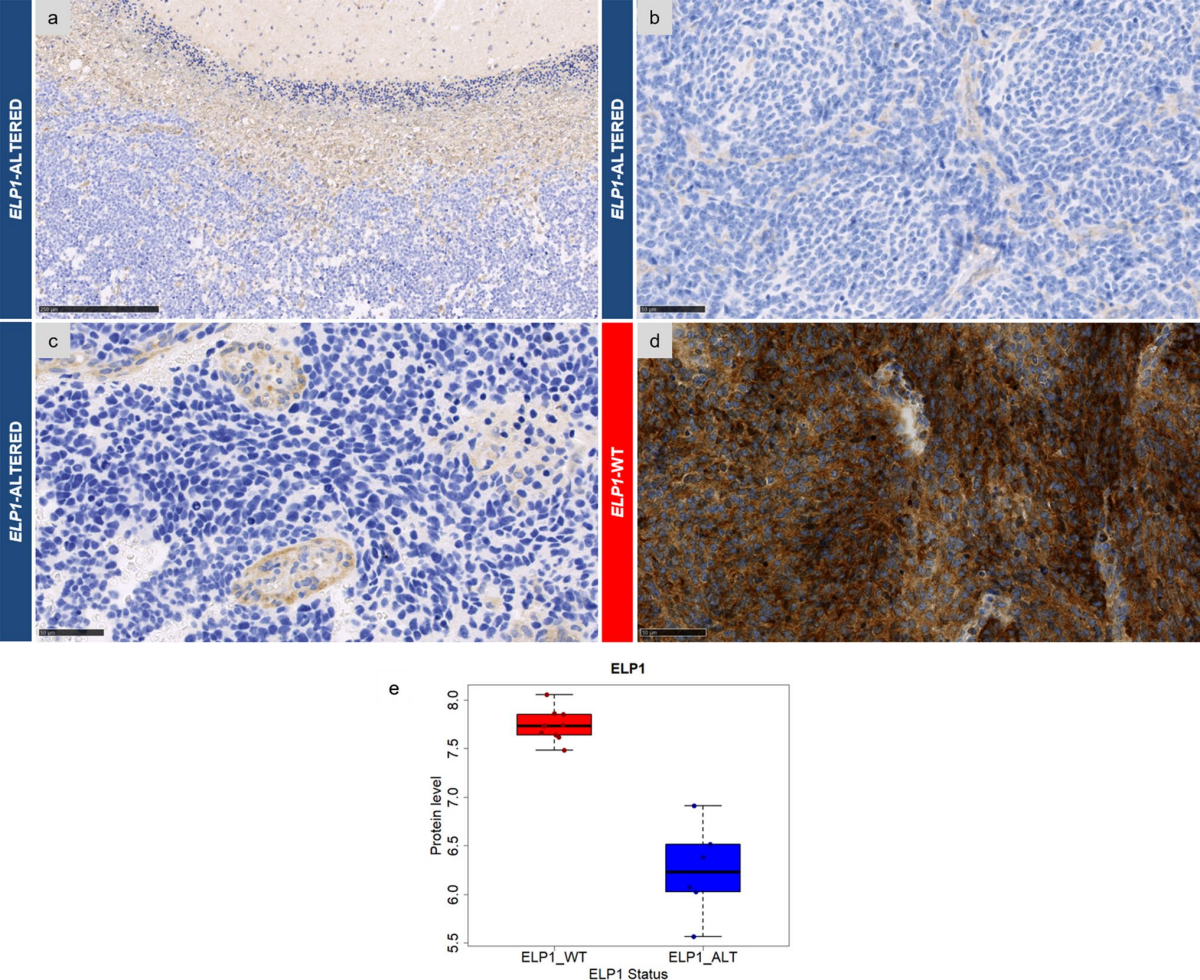
ELP1 expression in medulloblastomas SHH-activated. Case 1: A distinct ELP1 loss in one case of medulloblastoma, SHH-activated, with a bi-allelic alteration of *ELP1* with cerebellum parenchyma and vessels as positive internal controls (**a**, magnification, ×150). Case 1: A distinct ELP1 loss in one case of medulloblastoma, SHH-activated, with a bi-allelic alteration of *ELP1* (**b**, magnification, ×400, with endothelial cells as positive internal control). Case 7: A distinct ELP1 loss in one case of medulloblastoma, SHH-activated, with a bi-allelic alteration of *ELP1* (**c**, magnification, ×400). A preserved expression of ELP1 in one case of medulloblastoma, SHH-activated, without alteration of *ELP1* (**d**, magnification, ×400). Black scale bars represent 100 µm (**a**) and 50 μm (**b**–**d**)

**Table 1:** Histomolecular data of our cases with ELP1 loss of immuno-expression

Case number	Age at diagnosis (YO)	Histopathology	Molecular subgroup	IHC ELP1	SOMATIC ELP1 ALTERATION	PTCH1 status	9q status
1	2	EN	SHH-activated, TP53-WT	Lost	c.1622A > G/p.(Glu541Gly)	c.3131_3132insTGTCTTCCTGCTGAACCCCTGGACGGCCGGGATCAT/p.A1044delinsAVFLLNPWTAGII	Loss
2	5	D/N	SHH-activated, TP53-WT	Lost	c.2731C > T/p.(Gln911*)	c.3030dup/p.(Asn1011GlnfsTer134)	Loss
3	5	D/N	SHH-activated, TP53-WT	Lost	c.1461-2A > G/p.(His681Leu)	c.1225C > T/p.(Gln409Ter)	Loss
4	5	D/N	SHH-activated, TP53-WT	Lost	c.2731G > T/p.(Gln911Ter)	c.3030dup/p.(Asn1011GlnfsTer134)	Loss
5	5	D/N	SHH-activated, TP53-WT	Lost	c.676C > T/p.(Arg226Ter)	c.1197G > A/p.(Trp399Ter)	Loss
6	5	D/N	SHH-activated, TP53-WT	Lost	WT	c.2308C > T/p.(Arg770Ter)	Copy neutral-LOH
7	5	D/N	SHH-activated, TP53-WT	Lost	c.1229C > T / p.(Pro410Leu)	(p.Leu1086Ter)	Loss
8	6	D/N	SHH-activated, TP53-WT	Lost	c.3578delC / p.(Ser1193TyrfsTer30)	Somatic deletion	Loss
9	7	D/N	SHH-activated, TP53-WT	Lost	c.741-1G > T / p.(Glu1247Ter)	WT	Loss
10	8	D/N	SHH-activated, TP53-WT	Lost	c.1000C > T / p.(Gln334Ter)	WT	Loss
11	8	NOS (biopsy)	SHH-activated, TP53-WT	Lost	c.2499dup p.(Lys834Ter)	c.898del/p.(Ala300ProfsTer24)	Loss
12	9	D/N	SHH-activated, TP53-WT	Lost	c.961A > T / p.(His681ArgfsTer58)	WT	Loss

*D/N* Desmoplastic/nodular, *EN* extensive nodularity, *IHC* immunohistochemistry, *WT* wildtype, *YO* year-old

Altogether, ELP1 IHC constitutes a fast, low-cost and conservative tissue-consuming method to detect *ELP1-*associated MB. Only one case presented a loss of expression without a bi-allelic alteration of *ELP1* identified, suggesting the presence of a cryptic alteration (no deep intronic pathogenic variant, complex structural variant, promoter genomic alteration or hyper-methylation was detected with our NGS analysis)*.* The higher proportion (19 vs. 14% in the literature) is explained by the large number of children in our cohort [4]. Here, none of the group 4 MB (*n* = 30) harbored an *ELP1* mutation, confirming that this is a rare event, as already suggested by the previous literature [4].

To conclude, we demonstrated that ELP1 IHC is a highly specific and sensitive biomarker for identifying *ELP1-*associated MB and should be part of the neuropathologist’s routine panel of antibodies to possibly screen a related predisposition syndrome in these children.

## Supplementary Information

Below is the link to the electronic supplementary material.

Supplementary file1 (PDF 723 kb)

## Data Availability

Proteomic datasets were deposited to the Proteomics Identifications Database (PRIDE) with accession number PXD016832.
