# Supplementary material for: Immunohistochemistry as a tool to identify ELP1-associated medulloblastoma
Source: Acta Neuropathol. 2022 Feb 23;143(4):523–5. doi: 10.1007/s00401-022-02409-4 (PMC8960608; doi:10.1007/s00401-022-02409-4)
Supplement: Supplementary file 1 — Supplementary file1 (PDF 723 kb) [file 401_2022_2409_MOESM1_ESM.pdf]

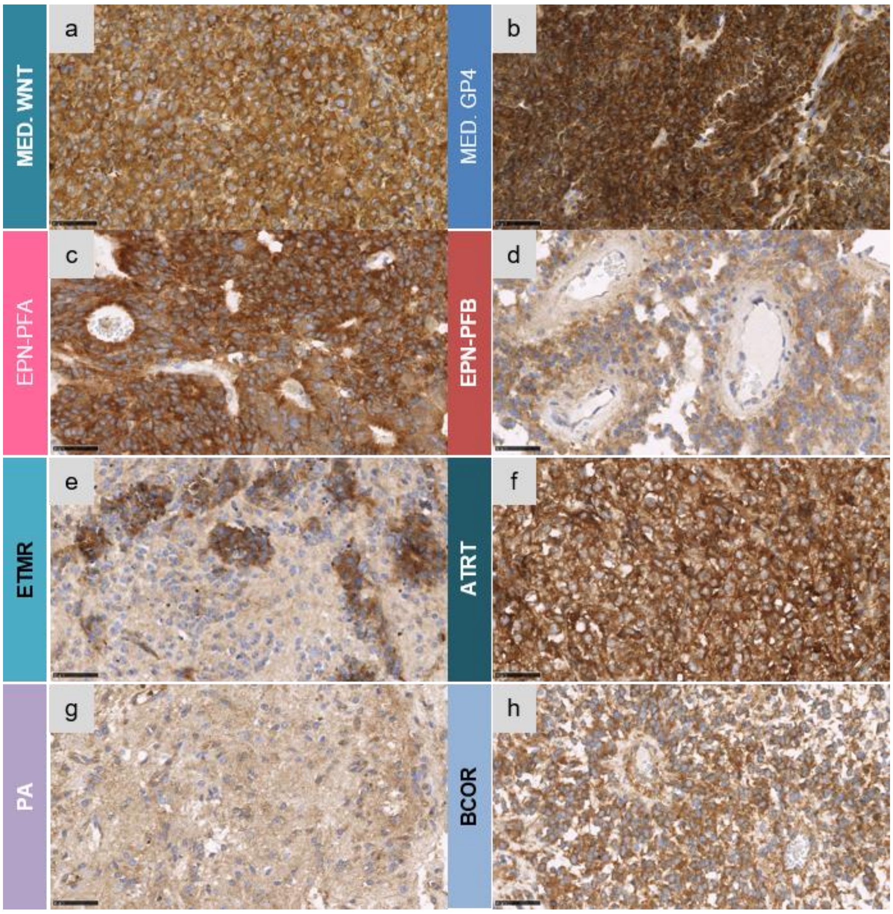

Supplementary Fig. 1 ELP1 expression in other groups of medulloblastomas and other tumors of the posterior fossa.

A preserved expression of ELP1 in all tested tumors (**a-h**, magnification, 400x).

ATRT: atypical teratoid and rhabdoid tumor; BCOR: central nervous tumor with *BCOR* internal tandem duplication; EPN: ependymoma; ETMR: embryonal tumor with multilayered rosettes; MED: medulloblastoma; PA: pilocytic astrocytoma; PFA: posterior fossa group A; PFB: posterior fossa group B; WT: wildtype.

Black scale bars represent 50 µm.

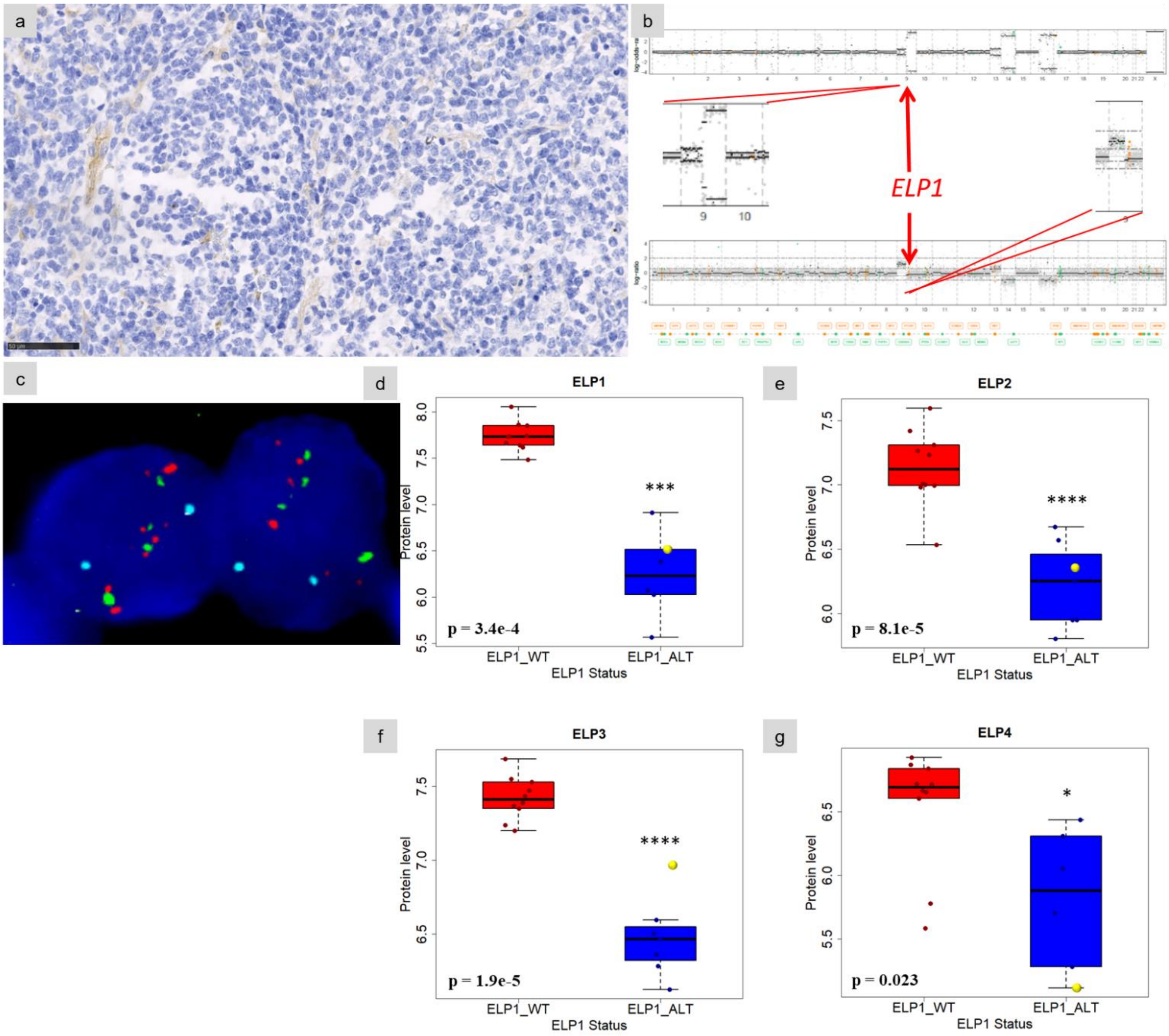

Supplementary Fig. 2 ELP1 expression in the discordant case (immunohistochemistry/molecular biology).

Loss of immunoexpression of ELP1 (**a**, magnification, 400x). A copy neutral loss of heterozygosity of chromosome 9q but no second alteration of *ELP1* found, **b**). FISH analysis showing gains of the locus CDKN2A (9p21, orange signals) and of the centromere of the chromosome 9 (9p11-9q11, green signals), with a disomy of the 9q locus (9q22, aqua signals) (**c**). Proteomic analysis of cases with downregulated protein expression of ELP1, ELP2, ELP3, and ELP4 (yellow dot) compared to medulloblastoma, SHH-activated, *ELP1*-altered and -wildtype (**d-g**).

ALT: altered; FISH : fluorescence in situ hybridization ; WT: wildtype.

Black scale bars represent 50 µm.

ABCB1	BCL10	CDC25C	DDX41	FAT1	HNF1A	MAP3K1	NFKBIE	PPP2R1A	RXRA	STAG2	WASF2
ABCG2	BCL11A	CDC27	DHX15	FAT2	HOXB13	MAPK1	NIPBL	PPP6C	SAMD9	STAT3	WASF3
ABHD5	BCL11B	CDH1	DICER1	FAT4	HRAS	MAX	NOTCH1	PRDM1	SAMD9L	STAT5B	WDR74
ABI1	BCL2	CDK12	DNAJC21	FBXO11	ID3	MBD4	NOTCH2	PREX2	SARAF	STAT6	WRN
ABI2	BCL2L1	CDK4	DNMT1	FBXW7	IDH1	MCL1	NOTCH3	PRIM2	SBDS	STK11	WT1
ABI3	BCL2L12	CDK6	DNMT3A	FGF10	IDH2	MDM2	NOTCH4	PRKCI	SDHA	STMN2	XPO1
ABL1	BCL6	CDK8	DPF1	FGF19	IGF1R	MDM4	NPM1	PRKD1	SDHB	SUFU	XRCC1
ACTL6A	BCL7A	CDKN1A	DPF2	FGF3	IGF2R	MECOM	NQO1	PRKD2	SDHC	SUZ12	XRCC2
ACTL6B	BCL7B	CDKN1B	DPF3	FGF4	IKBKAP	MED1	NR2F2	PRPF8	SDHD	SYF2	XRCC3
ACVR1	BCL7C	CDKN2A	DPYD	FGF6	IKZF1	MED12	NRAS	PRSS8	SETBP1	TAF1	ZDHHC19
ACVR1B	BCLAF1	CDKN2B	DROSHA	FGFR1	IKZF3	MED16	NSD1	PTCH1	SETD2	TAF1L	ZFHX3
ACVR2A	BCOR	CDKN2C	EBF1	FGFR2	IL6ST	MEF2B	NSD2	PTEN	SETDB1	TBC1D12	ZFP36L1
ACVR2B	BCORL1	CEBPA	EGFR	FGFR3	ING1	MEF2C	NT5C2	PTPN11	SF3B1	TBL1XR1	ZFP36L2
ADGRB3	BIRC2	CHD1	EIF1AX	FGFR4	INPP4B	MEN1	NTHL1	PTPRB	SF3B2	TBR1	ZIC1
ADGRG6	BIRC3	CHD2	ELANE	FH	IRF2	MET	NUMA1	PTPRD	SGK1	TBX3	ZMYM3
AGTR2	BLM	CHD3	ELF3	FLT1	IRF4	MFHAS1	NUP214	PTPRT	SH2B3	TCF12	ZMYM4
AJUBA	BMI1	CHD4	EMSY	FLT3	IRF8	MGA	NUP93	PXDNL	SLC1A2	TCF3	ZNF143
AKAP9	BMPR2	CHD6	EP300	FLT4	IRS2	miR-142	OTX2	QKI	SLC29A1	TCF4	ZNF292
AKT1	BRAF	CHD8	EP400	FOXA1	ITPKB	MITF	PALB2	RAB40A	SLC2A9	TCF7L2	ZNF471
AKT2	BRCA1	CHD9	EPHA2	FOXL2	JAK1	MLH1	PAX5	RAC1	SLIT2	TDG	ZNF750
AKT3	BRCA2	CHEK1	EPHA3	FOXO1	JAK2	MLH3	PAX6	RAD21	SLX4	TENT5C	ZRSR2
ALDOA	BRCC3	CHEK2	EPHA5	FOXP1	JAK3	MLLT4	PBRM1	RAD50	SMAD2	TERC	
ALK	BRD7	CIC	EPHA7	FOXR2	KANSL1	MN1	PCBP1	RAD51	SMAD3	TERT	
ALPK2	BRD9	CIITA	EPHB1	FRG1BP	KBTBD4	MPL	PDGFRA	RAD51B	SMAD4	TET1	
AMER1	BRIP1	CITED2	EPHB2	FRS2	KDM5C	MRE11	PDGFRB	RAD51C	SMARCA2	TET2	
ANKRD26	BRK1	CNOT9	EPPK1	FSHR	KDM6A	MSH2	PGR	RAD51D	SMARCA4	TET3	
APAF1	BTG1	COQ6	ERBB2	FUBP1	KDR	MSH3	PHF10	RAD54L	SMARCB1	TGFBR2	
APC	BTK	CRBN	ERBB3	GAS8-AS1	KEAP1	MSH6	PHF6	RAF1	SMARCC1	THAP12	
APC2	C19MC	CREBBP	ERBB4	GATA1	KIT	MST1R	PHGDH	RASA1	SMARCC2	THBS1	
AR	CALR	CRKL	ERBIN	GATA2	KLF2	MTHFR	PHOX2B	RASA2	SMARCD1	THSD7B	
ARAP3	CAND1.11	CSF1R	ERCC2	GATA3	KLF4	MTOR	PIK3C2B	RB1	SMARCD2	TLR4	
ARFRP1	CARD11	CSF3R	ESR1	GATA6	KLHL6	MUTYH	PIK3CA	RBBP6	SMARCD3	TNF	
ARHGAP35	CASP8	CSNK1A1	ETNK1	GFI1	KMT2A	MYB	PIK3CB	RBM10	SMARCE1	TNFAIP3	
ARID1A	CBFB	CTCF	ETV6	GLI1	KMT2B	MYC	PIK3CG	RECQL4	SMC1A	TNFRSF14	
ARID1B	CBL	CTNNA1	EXT1	GLI2	KMT2C	MYCL	PIK3R1	RET	SMC3	TP53	
ARID2	CBLB	CTNNB1	EXT2	GNA11	KMT2D	MYCN	PIK3R2	RHEB	SMO	TP53BP1	
ARID5B	CBLC	CTNND2	EZH2	GNA13	KRAS	MYD88	PIM1	RHOA	SMUG1	TP73	
ASXL1	CCDC107	CTPS1	FADD	GNAQ	LCK	MYO3A	PKD1	RICTOR	SNCAIP	TRAF2	
ASXL2	CCND1	CUL4B	FAM213A	GNAS	LDLRAP1	MYOD1	PLCB4	RIMS1	SNX25	TRAF3	
ATM	CCND2	CUX1	FANCA	GRIN2A	LEPROTL1	NAF1	PLCG1	RIT1	SOCS1	TRRAP	
ATR	CCND3	CXCR4	FANCB	GSTP1	LINC02008	NAV3	PLCG2	RMRP	SOX10	TSC1	
ATRX	CCNE1	CYFIP1	FANCC	H3F3A	LOC101927630	NBN	PLEKHS1	RNF169	SOX2	TSC2	
AURKA	CD274	CYFIP2	FANCD2	H3F3B	LYN	NCKAP1	PMS1	RNF43	SOX9	U2AF1	
AXIN1	CD28	CYP1A1	FANCE	HIST1H1B	LZTR1	NCKAP1L	PMS2	ROBO1	SPEN	U2AF2	

AXIN2	CD36	CYP2D6	FANCF	HIST1H3B	MALAT1	NCOR1	POLD1	ROBO2	SPOP	UBE2K
AXL	CD58	DAXX	FANCG	HIST1H3C	MALT1	NCOR2	POLE	ROS1	SRCAP	UBR5
B2M	CD70	DCK	FANCI	HIST2H3C	MAP2K1	NEAT1	POLE2	RPL22	SRP72	VEGFA
BAP1	CD79A	DCTD	FANCL	HLA-A	MAP2K2	NF1	POLR2D	RPL5	SRSF2	VHL
BARD1	CD79B	DDR2	FANCM	HLA-B	MAP2K4	NF2	POT1	RPTOR	SS18	WAS
BC040327	CDA	DDX3X	FAS	HLA-C	MAP2K7	NFE2L2	PPM1D	RUNX1	STAG1	WASF1

Supplementary Table. 1 List of genes examined by the custom Next generation sequencing (NGS) panel.
